# Editorial: Bioengineered gene and cell therapy for treating cardiovascular diseases

**DOI:** 10.3389/fbioe.2023.1250175

**Published:** 2023-08-01

**Authors:** Kai Wang, Xujie Liu, Xiaojun Lance Lian, Xiaoping Bao, Kailong Li

**Affiliations:** ^1^ State Key Laboratory of Vascular Homeostasis and Remodeling, Department of Physiology and Pathophysiology, School of Basic Medical Sciences, Peking University, Beijing, China; ^2^ State Key Laboratory of Cardiovascular Disease, Fuwai Hospital, Beijing, China; ^3^ Department of Biomedical Engineering, The Huck Institutes of the Life Sciences, Pennsylvania State University, University Park, PA, United States; ^4^ Department of Biology, The Huck Institutes of the Life Sciences, Pennsylvania State University, University Park, PA, United States; ^5^ Davdidson School of Chemical Engineering, Purdue University, West Lafayette, IN, United States; ^6^ Beijing Key Laboratory of Protein Posttranslational Modifications and Cell Function, Department of Biochemistry and Biophysics, School of Basic Medical Sciences, Peking University Health Science Center, Beijing, China

**Keywords:** maturation, cardiomyocytes, fibrosis, angiogenesis, cell therapy

Gene-and cell-based therapy applications show efficacy for the treatment of a variety of diseases, including cardiovascular diseases (Zhao et al., 2023). However, their therapeutic potential is currently not fully exploited due to several reasons. Firstly, delivering therapeutic cells or genetic material to the targeted site within the cardiovascular system can be challenging. It is crucial to ensure that the cells or genes reach the intended location and integrate effectively into the damaged tissues. Achieving precise targeting and efficient delivery remains a significant hurdle. Secondly, cell and gene therapies often involve the use of embryonic stem cells or gene-editing technologies. Ethical concerns related to the source of cells, manipulation of genetic material, and potential unintended consequences of gene editing need to be carefully addressed and monitored. Last but not least, ensuring long-term efficacy and durability of cell and gene therapies is a significant challenge. Transplanted cells may experience limited survival or fail to function optimally in the long run due to poor maturity.

Gene editing lay the foundation for creating novel gene therapy products, and the emergence of the clustered regularly interspaced short palindromic repeats (CRISPR)/Cas9 system has revolutionized genome editing due to its simplicity and effectiveness. The CRISPR/Cas9 system also has the inherent risk of unintended gene editing with similar DNA sequences and causes adverse effects. Of particular concern is that off-target effects might lead to the tumorigenicity of cells. Guo et al. gave an overview of how we can manage those off-target effects for better gene and cell therapy. Although multiple informative *in silico* prediction tools have been developed, the computational methods are usually biased towards sgRNA-dependent off-target effects that need experimental sequencing to reach consensus. Indeed, multiple sequencing methods including GUIDE-seq, Discover-seq, and Digenome-seq have been invented to quantify the off-targeting. In addition, several strategies for reducing the off-target effects are also summarized.

Fibrosis is mainly marked by the regression of parenchymal cells and progression of collagen-secreting fibroblasts. The excessive accumulation of collagen fibers disrupts the normal tissue structure and result in the loss of function in nearly all major organs, including the heart, lung, liver, and kidney. To counteract fibrosis, stem cell-derived therapies including pluripotent stem cells (PSC) have presented a promising approach since differentiated parenchymal cells could replace the loss. In a nicely organized review, Cheng et al. summarized the PSC-derived products, including a variety of cells and secreted vesicles, which all have the potential for alleviating fibrosis. Since adult stem cells such as mesenchymal stem cell (MSC) have long been applied for treating fibrosis, the authors make a direct comparison between MSC and PSC so that readers could have a sense of how to choose the right stem cells in different scenarios.

Myocardial infarction (MI) is an ischemic heart disease with significant loss of cardiomyocytes that could lead to fibrosis. Indeed, stem cell-derived cardiomyocytes hold promise for replenishing cell loss and ameliorating heart fibrosis (Hong et al., 2021). However, engrafted cardiomyocytes might cause ventricular arrhythmias due to their limited maturity. To gain more insights into this, Hong et al. wrote a systematic review on the cutting-edge bioengineering strategies for enhancing maturation. Those strategies consist of not only physical modulation, such as mechanical and electrical stimulation, but also biochemical modulation, including hormone and small molecules. In addition to those classical strategies, emerging methodologies, such as synthetic activation of vital transcription factors or metabolic programming, further accelerated maturation. One of the primary motivations behind enhancing maturation is to optimize engraftment. Intriguingly, the process of implantation itself also triggers maturation. This phenomenon entails intricate cellular communication and the establishment of a sophisticated microenvironment conducive to education, which proves challenging to replicate *in vitro*. Importantly, this *in vivo* maturation pattern extends beyond cardiomyocytes, encompassing stem cell-derived endothelial cells and pancreatic *β* cells as well (Wang et al., 2020; Wang et al., 2021).

In an original contribution, Zhan et al. reported on gene interference of vascular endothelial growth factor (VEGF) to dampen tumor angiogenesis in order to promote the homogenous distribution of tumor blood vessels (Zhang et al., 2020). The normalization of the vascular microenvironment further boosted tumor immunotherapy mediated by the anti-PD-L1 antibody. This combination therapy might offer a new avenue for clinical tumor immunotherapy. An important aspect to highlight is the utilization of hyaluronic acid/polyethyleneimine/CpG nanoparticles, which effectively facilitated the targeted delivery of small hairpin RNA against VEGF into the tumor.

Collectively, precise and advanced gene and cell therapy approaches could enrich the pipelines for combating cardiovascular diseases. The manuscripts in this Research Topic, comprising three review papers and one original contribution from a diverse set of authors, could be a valuable addition to the field.
